# A Simple Condition Monitoring Method for Gearboxes Operating in Impulsive Environments

**DOI:** 10.3390/s20072115

**Published:** 2020-04-09

**Authors:** Stephan Schmidt, Radoslaw Zimroz, Fakher Chaari, P. Stephan Heyns, Mohamed Haddar

**Affiliations:** 1 Centre for Asset Integrity Management, Department of Mechanical and Aeronautical Engineering, University of Pretoria, Pretoria 0002, South Africa; stephan.heyns@up.ac.za; 2 Faculty of Geoengineering, Mining and Geology, Wroclaw University of Science and Technology, Na Grobli 15, 50-421 Wroclaw, Poland; radoslaw.zimroz@pwr.edu.pl; 3 Laboratory of Mechanics, Modelling and Production, National School of Engineers of Sfax, BP1173, Sfax 3038, Tunisia; fakher.chaari@gmail.com (F.C.); mohamed.haddar2016@gmail.com (M.H.)

**Keywords:** gearbox diagnostics, synchronous median of the squared envelope, time-varying operating conditions

## Abstract

Reliable condition indicators are necessary to perform effective diagnosis and prognosis. However, the vibration signals are often corrupted with non-Gaussian noise and rotating machines may operate under time-varying operating conditions. This impedes the application of conventional condition indicators. The synchronous average of the squared envelope is a relatively simple yet effective method to perform fault detection, fault identification and fault trending under constant and time-varying operating conditions. However, its performance is impeded by the presence of impulsive signal components attributed to impulsive noise or the presence of other damage modes in the machine. In this work, it is proposed that the synchronous median of the squared envelope should be used instead of the synchronous average of the squared envelope for gearbox fault diagnosis. It is shown on numerical and experimental datasets that the synchronous median is more robust to the presence of impulsive signal components and is therefore more reliable for estimating the condition of specific machine components.

## 1. Introduction

Condition-Based Maintenance (CBM) is needed to ensure that expensive assets such as wind turbines can perform reliably and cost-effectively. In CBM, the condition of the machine is estimated with fault diagnosis techniques from the available condition monitoring data, whereafter prognosis techniques are applied to estimate the remaining useful life of the machine [1]. The estimated condition of the machine is used as a basis for maintenance decisions, which can make CBM more cost-effective than conventional time-based preventative maintenance approaches [1,2]. However, the performance of CBM depends on the ability of the fault diagnosis techniques to accurately identify the condition of the different mechanical components while the machine is operating under its normal operating conditions. Many machines found in the power generation and mining industries operate inherently under time-varying operating conditions, which impede the performance of conventional fault diagnosis techniques and therefore reliable techniques are required to perform fault diagnosis, i.e., fault detection, fault localisation and fault trending [3,4].

Gearboxes are critical components in many industries and are subjected to harsh operating conditions which make them susceptible to damage and failure, with long downtimes associated with each replacement [5,6]. Localised gear damage and distributed gear damage modes are frequently encountered in gearboxes and therefore need to be detected and characterised early to ensure that the appropriate maintenance decisions can be made [7]. It is important to determine the damage mode, e.g., localised or distributed gear damage, because this influences the remaining useful life of the machine component, e.g., localised gear damage is expected to deteriorate fast due to the localised increase in stress [8].

Gear damage such as fatigue cracks can excite resonance bands and result in the cyclostationary content of the vibration signals to increase. Any phase shifts during the measurement period can also impede the application of the conventional synchronous average [9] and therefore the Synchronous Average of the Squared Envelope (SASE) is better suited. The SASE has been used for gear and bearing diagnostics [10,11,12,13]. Schmidt and Heyns [14] developed a localised anomaly detection methodology by comparing the probability density functions of the synchronous statistics of different gear teeth with the Kullback–Leibler divergence.

The excitation of structural resonances by the impacts of damage components manifest in time-invariant frequency bands [15], with frequency band identification techniques such as the kurtogram [16] and the IFBI${}_{\alpha }$gram [12] making it possible to automatically determine the frequency bands that are rich with impulsive information [17,18]. Schmidt et al. [13] combined frequency band identification methods with healthy historical data to identify frequency bands with novel information, i.e., due to damage, whereafter the SA and SASE of the filtered signal are calculated for detecting and visualising the gear damage. The SASE performed better than the SA for incipient fault detection.

The SASE has also been used in the derivation of condition indicators that can be used for hypothesis testing when performing condition inference [19]. If the hypothesis of a cyclostationary signal buried in Gaussian stationary noise is tested against the presence of only Gaussian stationary noise, the test statistic is a function of the SASE [19]. This has significant benefits due to the simplicity of the indicator and the possibility to design condition indicators for detecting the damage of specific mechanical components.

Bartelmus and Zimroz [4] and Zimroz et al. [3] developed methods to obtain robust condition indicators under time-varying operating conditions by conditioning indicators such as the root-mean-square on the operating conditions. The method is simple to implement and has performed well under time-varying operating conditions [3,4]. However, those methods do not allow the changes to be ascribed to a specific mechanical component. Extracting features from the SASE would theoretically make it possible to determine which component is damaged. However, it is shown in this work that the

- SASE is sensitive to non-Gaussian noise.
- SASE is not capable of separating the synchronous and non-synchronous damaged components.

Hence, in this paper, we propose that the Synchronous Median of the Squared Envelope (SMSE) should be used instead of the SASE for inferring the condition of rotating machine components. This subtle change (i.e., using the median statistic as opposed to the mean statistic) has significant benefits when performing condition monitoring on rotating machines. This is because even if the rotating machine under consideration does not operate in impulsive noise environments, non-synchronous damaged components (e.g., a damaged bearing when the pinion is interrogated) impede the performance of the SASE, but not the performance of the SMSE. This is attributed to the fact that the median is a more robust measure of the central tendency of a random variable than the mean.

The layout of this paper is as follows: In Section 2, an overview of the SASE and SMSE are given for fault diagnosis and using it for performing fault diagnosis under time-varying operating conditions. In Section 3, the SASE and SMSE are compared on numerical data, whereafter the two statistics are compared in Section 4 on experimental data. The work is finally concluded in Section 5. In Appendix A, supporting information is provided for the motivation of the SMSE in Section 2.

## 2. Gearbox Diagnostics

The acquired vibration data contain information related to the machine and its operating environment. This can typically include the interaction of healthy machine components, e.g., excitations during gear meshing; the influence of damage on the component-of-interest, e.g., impulses of a damaged bearing; the potential presence of other damaged components or other damage modes; and the influence of time-varying operating conditions. Since, the mechanical components have fixed kinematics that govern their movement, the vibration signals are cyclostationary in the angle domain, with the cyclostationarity in the time domain being a special case [20]. Hence, the time domain signal is transformed to an angle domain representation using order tracking to ensure that the impulses are periodic [21,22].

**Figure 1:**
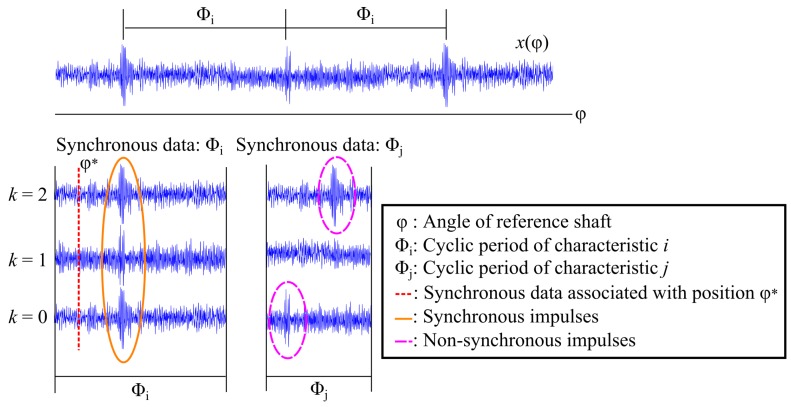
A vibration signal is shown with impulses that have a cyclic period of $\Phi_{i}$, i.e., a cyclic order of $\Phi_{i}^{-1}$, and a synchronous representation is shown for two cyclic periods $\Phi_{i}$ and $\Phi_{j}$. The rotation number is denoted *k*.

The SA and the SASE have been successfully used for gear and bearing diagnostics [10,11,13]. This is because the damaged components are periodic with some period $\Phi_{i}$ and would therefore be retained when calculating the SA and/or the SASE. The SA is shown in Figure 1 for two components being monitored.

If sufficient rotations are considered then the non-synchronous impulses manifesting in the SA associated with $\Phi_{j}$ will be attenuated, while the synchronous impulses in SA associated with $\Phi_{i}$ will be retained. However, the phase-shifts encountered under time-varying operating conditions can impede the performance of the SA with some of the diagnostic information attenuated [9] and therefore the SASE is considered instead.

The SASE is better suited than the SA for incipient fault detection [13], because small phase changes do not lead to the diagnostic information to be attenuated and impulses typically manifest as second-order cyclostationary components. However, by using the SASE, it also makes the related diagnostic metrics more sensitive to impulsive non-synchronous components. It is desired to exploit the sensitivity of the SASE to incipient damage, without it being so sensitive to non-synchronous components. This is investigated by firstly understanding the statistical properties of the synchronous data of the squared envelope, referred to only as synchronous data throughout the rest of the paper.

### 2.1. Synchronous Data Modelling

We assume that the squared envelope ${\left|x\left(\varphi +k\cdot \Phi_{i}\right)\right|}^{2}$ at an angular position $\varphi +k\cdot \Phi_{i}$ is generated by

(1) $${\left|x\left(\varphi +k\cdot \Phi_{i}\right)\right|}^{2}∼p\left({\left|x\left(\varphi \right)\right|}^{2};\varphi ,\Phi_{i}\right),\;\mathrm{for}\;k\in Z,$$

where $p({\left|x\left(\varphi \right)\right|}^{2};\varphi ,\Phi_{i})$ is a distribution that is dependent on the angular position $\varphi \in [0,\Phi_{i})$ and the cyclic period under consideration $\Phi_{i}$. This assumption is appropriate for stationary operating conditions or time-varying operating conditions that result in small changes in the amplitude of the signals. This approximation can be extended to general time-varying operating conditions when a normalised signal, obtained with a Normalisation of the Amplitude Modulation due to Varying Operating Conditions (NAMVOC) method [23], is considered instead of the raw signal.

The signal $x\left(\varphi \right)$ is typically non-Gaussian, because it can contain impulses of multiple damage modes and impulsive noise. These characteristics need to be considered when selecting a statistic that is used for fault detection. The mixture model

(2) $$p\left({\left|x\left(\varphi \right)\right|}^{2};\varphi ,\Phi_{i}\right)=w\cdot p\left({\left|x\left(\varphi \right)\right|}^{2};\theta_{1},\varphi ,\Phi_{i}\right)+\left(1-w\right)\cdot p\left({\left|x\left(\varphi \right)\right|}^{2};\theta_{2},\varphi ,\Phi_{i}\right),$$

is used to investigate the robustness of the estimators in this work. For the purposes of the subsequent investigations, it is assumed that in Equation (2), the component-of-interest is healthy, where *w* is its weight and $p({\left|x\left(\varphi \right)\right|}^{2};\theta_{1},\varphi ,\Phi_{i})$ is the probability density function of its generated data with parameters $\theta_{1}$. The parameters $\theta_{1}$ can, for example, contain the mean $\mu_{1}$ of the probability density function.

**Figure A1:**
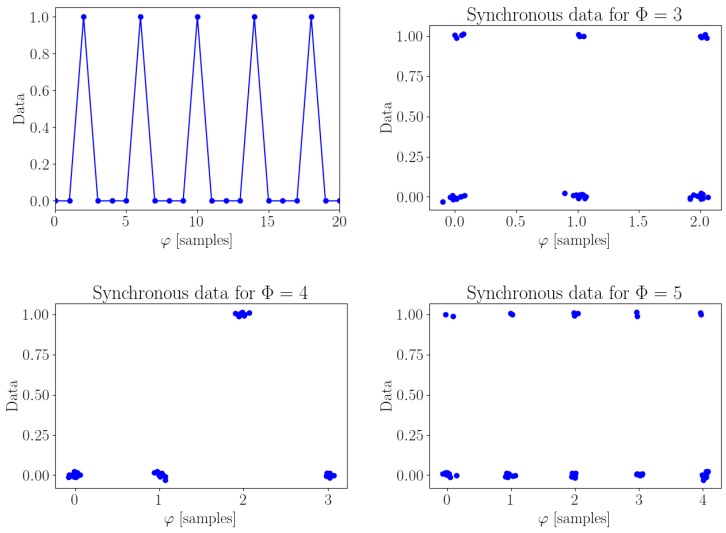
The potential multimodality of the synchronous data is presented for a synthetic signal and three cases of Φ. The data are jittered to make it easier to see the multimodal characteristics.

The second component in Equation (2) is attributed to damage that is non-synchronous with the period under consideration. It is weighted by (1−w) and is described by the probability density function $p({\left|x\left(\varphi \right)\right|}^{2};\theta_{2},\varphi ,\Phi_{i})$ with parameters $\theta_{2}$. A mixture model is very useful to investigate the robustness of estimators when a small fraction of the data is contaminated [24,25]. The fraction of contamination is given by (1−w) and is used to represent the impulses generated by the damaged non-synchronous components. This mixture model is reasonable as shown in Figure A1 in Appendix A. The non-synchronous impulses lead to the synchronous data to display multimodal characteristics.

### 2.2. Estimating the Central Tendency of the Synchronous Data

The central tendency of the squared envelope provides an estimate of the instantaneous power that is generated by the damaged component. If a robust estimate of the central tendency is obtained, it can be used to estimate the strength of the impulses in the vibration signal, which can subsequently be used to infer the condition of the machine. However, care should be taken to directly correlate the magnitude of the amplitude with the severity of the fault, because some fluctuations in the impulse magnitude are expected as the component degrades. El-Thalji and Jantunen [26] provided a good explanation on why this phenomenon occurs during the life of bearings.

The expected value of ${\left|x\left(\varphi \right)\right|}^{2}$ for a specific angular period $\Phi_{i}$

(3) $$E\left\{{\left|x\left(\varphi \right)\right|}^{2};\Phi_{i}\right\}=\int {\left|x\left(\varphi \right)\right|}^{2}\cdot p({\left|x\left(\varphi \right)\right|}^{2};\Phi_{i})d{\left|x\left(\varphi \right)\right|}^{2},$$

can be used as a measure of the central tendency of ${\left|x\left(\varphi \right)\right|}^{2}$, which can also be calculated with

(4) $$E\left\{{\left|x\left(\varphi \right)\right|}^{2};\Phi_{i}\right\}=\lim_{N_{\Phi_{i}}\to \infty }\frac{1}{N_{\Phi_{i}}}\sum_{k=0}^{N_{\Phi_{i}}-1}{\left|x\left(\varphi +k\cdot \Phi_{i}\right)\right|}^{2},$$

where ${\left|x\left(\varphi +k\cdot \Phi_{i}\right)\right|}^{2}$ is generated with Equation (1) and *k* is shown in Figure 1. The expected value of the mixture model in Equation (2),

(5) $$E\left\{{\left|x\left(\varphi \right)\right|}^{2};\Phi_{i}\right\}=w\cdot \mu_{1}+\left(1-w\right)\cdot \mu_{2},$$

is a function of both the mean of the component-of-interest $\mu_{1}$ and the mean of the non-synchronous data $\mu_{2}$. If the non-synchronous component is attributed to another damage component, $\mu_{2}$ will change over time as the component deteriorates. This means that the average, i.e., $E\{{\left|x\left(\varphi \right)\right|}^{2}\}$, cannot be used to distinguish between changes in the component-of-interest, i.e., $\mu_{1}$, and changes in the non-synchronous component, i.e., $\mu_{2}$.

This is a well known result when considering robust estimators; the average is only a very efficient estimator if the data are generated by a Gaussian distribution [24,25]. If the data are contaminated, more robust estimators such as the median need to be used. The median of the synchronous data at an angle φ, denoted $\mathrm{med}\{{\left|x\left(\varphi \right)\right|}^{2};\Phi_{i}\}$, can be obtained by solving

(6) $$0.5=F_{cdf}\left(\mathrm{med}\left\{{\left|x\left(\varphi \right)\right|}^{2};\Phi_{i}\right\}\right),$$

where $F_{cdf}$ is the cumulative distribution corresponding to the probability density function $p({\left|x\left(\varphi \right)\right|}^{2};\varphi ,\Phi_{i})$. The median of the mixture model considered in Equation (2) can be written to be dependent on only the first mode

(7) $$\mathrm{med}\left\{{\left|x\left(\varphi \right)\right|}^{2}\right\}=F\left(w,p\left({\left|x\left(\varphi \right)\right|}^{2};\theta_{1},\varphi ,\Phi_{i}\right)\right),$$

for some functional F, if the following assumptions are made:

- w>0.5. This is a reasonable assumption because it is expected that the synchronous characteristics would be more representative than the non-synchronous data at a specific angular position.
- The distributions are non-overlapping. This assumption is made for mathematical convenience, but is also the most difficult case to consider. This is because the damage of the non-synchronous component is then very prominent in the data and would likely affect the estimated statistics.

Equation (7) indicates that if the aforementioned assumptions are valid, then the median is only a function of the probability density function of the component-of-interest and not dependent on the magnitude of the non-synchronous component, i.e., it is not sensitive to any changes in $\mu_{2}$ and therefore provides a more reliable estimate of the component-of-interest than the average.

### 2.3. Synchronous Statistics

If the synchronous data over a period of $\Phi_{i}$, associated with angular position φ, are written in vector form,

(8) $${\left|x\left(\varphi ;\Phi_{i}\right)\right|}^{2}=\left[{\left|x\left(\varphi \right)\right|}^{2},\;{\left|x\left(\varphi +1\cdot \Phi_{i}\right)\right|}^{2},\ldots ,{\left|x\left(\varphi +N_{\Phi_{i}}\cdot \Phi_{i}\right)\right|}^{2}\right],$$

where $N_{\Phi_{i}}=\left\lfloor \max\left\{\varphi \right\}/\Phi_{i}\right\rfloor$, then the SASE is defined as

(9) $$s^{2}\left(\varphi ;\Phi_{i}\right)=\mathrm{average}\left({\left|x\left(\varphi ;\Phi_{i}\right)\right|}^{2}\right),$$

and the SMSE is defined as

(10) $$m^{2}\left(\varphi ;\Phi_{i}\right)=\mathrm{median}\left({\left|x\left(\varphi ;\Phi_{i}\right)\right|}^{2}\right).$$

The SASE in Equation (9) can also be calculated by setting the $N_{\Phi_{i}}\to \infty$ in Equation (4) to a finite value, i.e., $N_{\Phi_{i}}=\left\lfloor \max\left\{\varphi \right\}/\Phi_{i}\right\rfloor$. This corresponds then to the well-known form of the SASE.

### 2.4. Condition Indicators

Statistical and signal processing methods [19,27], statistical learning methods [28] and machine and deep learning methods [29,30] have been used to obtain condition indicators for rotating machines. Simple condition indicators such as the RMS are frequently used in diagnosis and prognosis applications, with the RMS being one of the most popular condition indicators for prognosis [1]. However, many of the simple one-dimensional metrics can only indicate if the condition of the machine changes, and cannot be used to infer the condition of the individual components that make up the machine. The synchronous representations of the condition of the machines (e.g., SASE, SMSE) could however be processed into one-dimensional metrics to not only detect the presence of damage, but also to detect changes in the condition of specific components of the machine. Different statistics of the SASE and SMSE could be extracted such as the mean and kurtosis [11,13]. Antoni and Borghesani [19] developed a methodology to derive condition indicators for condition monitoring based on the statistical properties of the vibration signals and derived

(11) $$I_{SASE}\left(\Phi_{i}\right)=-\langle \log\left(\frac{s^{2}\left(\varphi ;\Phi_{i}\right)}{\langle s^{2}\left(\varphi ;\Phi_{i}\right)\rangle }\right)\rangle ,$$

to test the Gaussian cyclostationary hypothesis against a Gaussian stationary hypothesis, with <·> denoting the time-average operator and $s^{2}\left(\varphi ;\Phi_{i}\right)$ the SASE. This metric can be used for trending the components associated with a period of $\Phi_{i}$ radians, which is very important when the damaged component needs to be identified.

Since, we propose that the SMSE should be used instead of the SASE, we replace the SASE in Equation (11) with the SMSE to obtain

(12) $$I_{SMSE}\left(\Phi_{i}\right)=-\langle \log\left(\frac{m^{2}\left(\varphi ;\Phi_{i}\right)}{\langle m^{2}\left(\varphi ;\Phi_{i}\right)\rangle }\right)\rangle .$$

In contrast to the work in Ref. [19], we are not using our indicators in any statistical tests and therefore we did not derive the statistical properties of the estimator given by Equation (12). Since the SMSE is better suited for impulsive noise and non-synchronous damaged components, it means that the condition indicator using the SMSE (i.e., Equation (12)) would be better suited for damage detection when compared to the SASE.

However, many rotating machines operate under time-varying operating conditions, which could impede the performance of the aforementioned condition indicators to deal with this. Bartelmus and Zimroz [4] and Zimroz et al. [3] proposed that the metric should be compared against the operating conditions of the machine. Hence, the conditional condition indicator $I_{SMSE}\left(\Phi_{i}\right)|\omega$ would be more robust to time-varying operating conditions than the unconditional condition indicator $I_{SMSE}\left(\Phi_{i}\right)$. We therefore propose that the conditional condition indicator, i.e., $I_{SMSE}\left(\Phi_{i}\right)|\omega$ should be used if the operating conditions are different for each measurement. This is illustrated in Section 4.3.

The suitability of the SMSE and SASE is investigated on numerical data in the next section.

## 3. Numerical Investigation

In this section, it is desired to compare the suitability of the SASE and SMSE for fault diagnosis in the presence of impulsive noise. This is performed by comparing the robustness of the average and the median on noise with different levels of impulsiveness. Thereafter, the SMSE and SASE are compared on a synthesised signal to determine whether they can distinguish between completely random and periodic impulses. In the next section, modelling impulsive noise with an α-stable distribution is discussed.

### 3.1. Modelling Impulsive Noise: α-Stable Processes

Impulsive noise, which can, for example, manifest due to the presence of electromagnetic interference [31], other damage modes [32], or due to the crushing process in a copper-ore crusher [33], needs to be considered when investigating the robustness of the SASE and SMSE for fault diagnosis. Alpha-stable distributions have been used in Refs. [34,35,36] to model stochastic impulsive noise signals in gearboxes and can also be used to investigate the robustness of different metrics for different levels of impulsiveness.

The probability density function of an α-stable distribution is given by

(13) $$p\left(r\right)=\frac{1}{2\pi }\int_{-\infty }^{\infty }\psi \left(y\right)e^{-iry}dy,$$

where the characteristic function ψ(y) is given by [34]

(14) $$\psi \left(y;\alpha ,\beta ,\sigma ,\mu \right)=\exp\left(-\sigma^{\alpha }\cdot {\left|y\right|}^{\alpha }\cdot \left(1+i\cdot \beta \cdot \mathrm{sgn}(y)\cdot B(y,\alpha )\right)+i\cdot \mu \cdot y\right),$$

with $i^{2}=-1$, α describes the impulsiveness of the generated data, β describes its skewness, σ is a dispersion parameter and μ is the mean of the data. The function B(y,α) is given by

(15) $$B\left(y,\alpha \right)=\left\{\begin{array}{ccc} \frac{2}{\pi }\ln\left|y\right| & \;\mathrm{if}\; & \alpha =1,\\ -\tan\left(\pi \alpha /2\right) & \;\mathrm{if}\; & \alpha \neq 1. \end{array}\right.$$

The most important parameter of the α-stable distribution is the α parameter, which describes the impulsiveness of the signal. If α=2, the generated samples follow a Gaussian distribution and as α decreases, the impulsiveness of the signal increases. The performance of the average and the median are compared for different impulsiveness levels, i.e., different α. The data are generated by a zero-mean, symmetric distribution, which is obtained by setting β=0, σ=1, and μ=0, and denoted by ASN(α) throughout the rest of the paper.

### 3.2. Convergence of the Average and Median Estimators

The samples generated by the α-stable distribution

**Figure 2:**
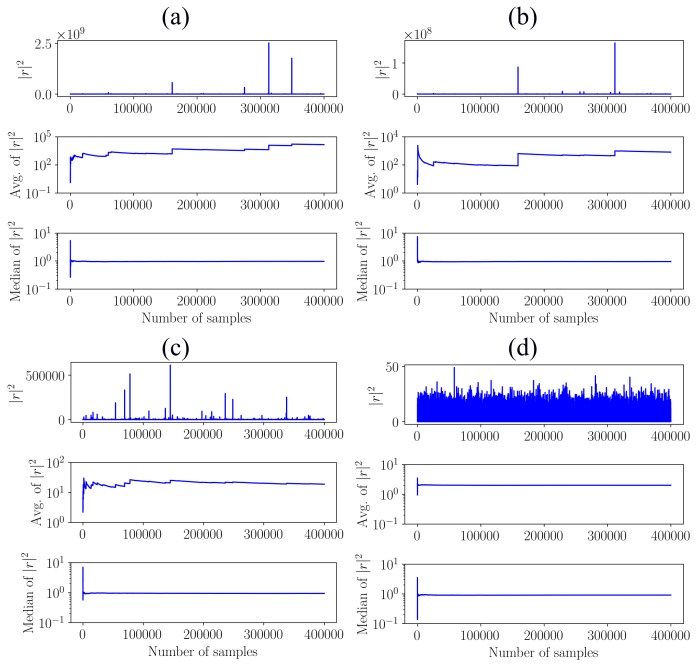
The mean and median of ${\left|r\right|}^{2}$ are shown as functions of sample number. The variable *r* is sampled from an α-stable distribution with α shown in the titles of the figures. If the sample number is *n*, it means that the first *n* samples are used to either calculate the mean or the median. (**a**) α=1.2; (**b**) α=1.4; (**c**) α=1.6; (**d**) α=2.0.

(16) r∼ASN(α),

for a specific α is identically and independently distributed, and because it is purely random, i.e., it is not periodic, it should not affect the performance of a robust estimator of the central tendency. Hence, the convergence of the mean and the median is investigated for the squared α-stable noise ${\left|r\right|}^{2}$ as a function of different α values by presenting the statistics as a function of number of samples that are used to calculate the statistics in Figure 2.

The average or mean is severely affected by impulses as seen in Figure 2a–c, which either results in a diverging metric (Figure 2a,b) or a very slow convergence (Figure 2c). The average only converges fast if the noise *r* is Gaussian, i.e., α=2.0. This is attributed to the fractional lower order statistical property of α-stable distributions, where the expected value of ${\left|r\right|}^{p}$ [34]

(17) $$E\{{\left|r\right|}^{p}\}\to \infty ,\;\mathrm{for}\;\alpha <p,$$

if 0<α<2. This indicates that the average is ill-suited for estimating the characteristics of squared non-Gaussian noise and therefore indicates that the SASE is ill-suited for fault diagnosis in impulsive environments. In contrast, the median is significantly robust to the impulsive behaviour of *r* with very similar convergence characteristics seen for all the considered α values.

### 3.3. Performance of SMSE and SASE in Impulsive Noise

In this section, it is assumed that the vibration generated by a damaged gear is generated by a second-order cyclostationary component with a cyclic period of $\Phi_{i}=2\pi$ rad, while a healthy gear with a cyclic period of $\Phi_{1}=40\pi /37$ shaft rotations is also present. The cyclic periods correspond to a cyclic frequency of 1.0 and 1.85 shaft orders, which are selected the same as the cyclic periods of the gear and pinion in the experimental setup presented in Section 4.1. The purpose of this investigation is to see whether it is possible to detect the damage gear component and to infer that the second gear is healthy in the presence of different noise distributions.

The acquired vibration signal is generated by

(18) x(φ)=y(φ)+r(φ),

where y(φ) is a second-order cyclostationary signal in the form

(19) $$y\left(\varphi \right)=V_{1}\cdot \sum_{k=0}^{N-1}\exp\left(-V_{2}\cdot \left(\varphi -k\cdot \Phi_{0}\right)\right)\cdot \epsilon \left(\varphi \right),$$

where ϵ(φ) is a sample from a zero-mean Gaussian distribution with unit variance and r(φ) is sampled from an α-stable distribution with Equation (16). The cyclic period of the signal is denoted by $\Phi_{0}$. The parameter $V_{1}$ scales the amplitude of the signal component and is set to 10 and the parameter $V_{2}$ scales the duration of the impulse (i.e., how long the impulse lasts) and is set to 40.

**Figure 3:**
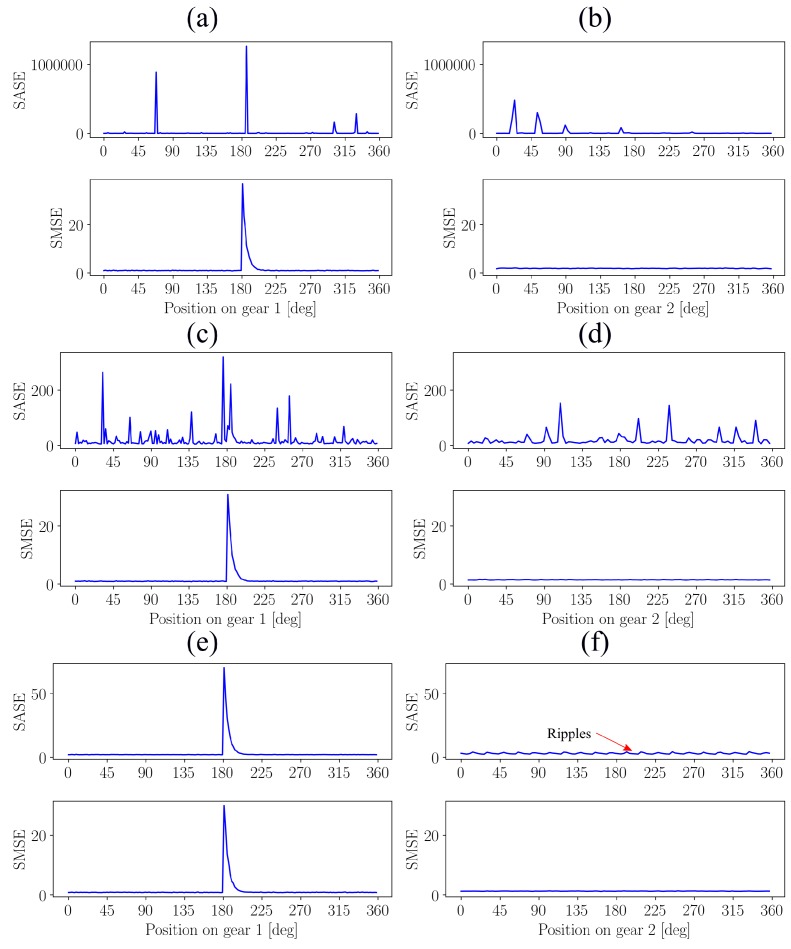
The SASE and the SMSE of the synthetic signal generated with Equation (18). Three levels of impulsiveness and two gears are considered, with gear 1 being damaged and gear 2 being healthy. The typically amplitudes of the squared noise data of different αs are shown in Figure 2. (**a**) Gear 1: α=1.2; (**b**) Gear 2: α=1.2; (**c**) Gear 1: α=1.6; (**d**) Gear 2: α=1.6; (**e**) Gear 1: α=2.0; (**f**) Gear 2: α=2.0

The acquired vibration signal is generated for a fixed damaged component y(φ), but with noise generated from α-stable distributions that have different αs. The SASE and SMSE are presented in Figure 3 for the investigated cases, with the damage impulse located at 180 degrees for the first gear, i.e., gear 1.

The SASE is adversely affected by the impulsive noise for α<2; the component attributed to the gear damage cannot be seen in Figure 3a,c, while it is also not possible to determine the condition of the second gear in Figure 3b,d. This is attributed to the sensitivity of the SASE to impulsive noise. However, if α=2, Gaussian noise is present and then it is possible to determine the condition of the gear with the SASE as seen in Figure 3e. However, when investigating the SASE of the second gear in Figure 3f, ripples can be seen. These ripples are attributed to the damage component associated with the first gear that manifest in the SASE of the second gear. This indicates that the SASE of the gear and the pinion are not independent from one another as indicated by Equation (5).

The SMSE performs much better than the SASE, because the gear damage can be detected for each considered α as seen in Figure 3a,c,e. In Figure 3b,d,f, it is also seen that the second gear does not contain any indication of being damaged and is clearly not influenced by the noise and the non-synchronous damaged component. Hence, the SMSE provides a more reliable estimate of the condition of the components despite the presence of very impulsive noise.

In the next section, the SASE and SMSE are investigated on experimental data.

## 4. Experimental Investigation

In this section, the suitability of SMSE and the SASE are compared on experimental data that were acquired under time-varying operating conditions. In the next section, an overview of the experimental setup is given, whereafter the results for a gear with localised damage and a gear with distributed damage are presented in Section 4.2 and Section 4.3 respectively.

### 4.1. Overview of Experimental Setup

**Figure 4:**
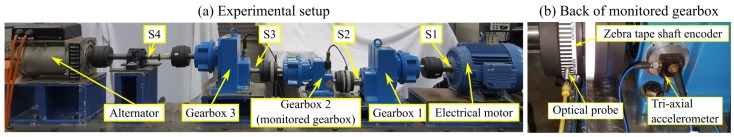
The experimental setup that was used to generate the datasets. In (**a**) the main components are highlighted and in (**b**) the sensors located on the back of the monitored gearbox are highlighted.

The experimental data considered in this section were measured on the experimental setup shown in Figure 4. This experimental setup is located in the Centre for Asset Integrity Management laboratory at the University of Pretoria and consists of three helical gearboxes, an electrical motor and an alternator which are highlighted in Figure 4a. The electrical motor drives the system and the alternator dissipates the rotational energy from the system and can be used to induce time-varying speed and load conditions.

**Figure 5:**
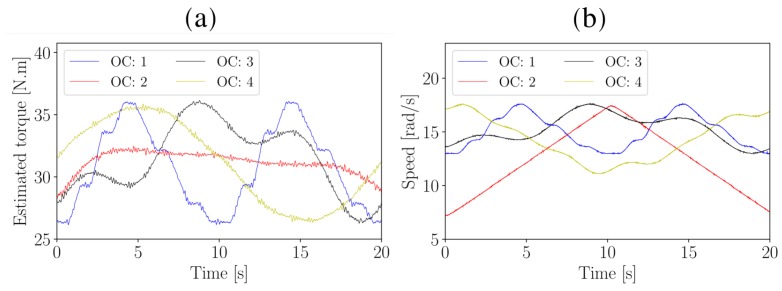
The load and the speed at the input shaft of the test gearbox. (**a**) Estimated torque for the four operating conditions; (**b**) Rotational speed profile for the four operating conditions.

The vibration of the test gearbox is measured with a tri-axial accelerometer located on the back of the gearbox as shown in Figure 4b. The axial channel of the tri-axial accelerometer is used for monitoring the condition of the helical test gearbox. The instantaneous rotational speed of the input shaft is measured with the optical probe and zebra-tape shaft encoder shown in Figure 4b. The instantaneous operating conditions that were present during the experiments are shown in Figure 5.

The gearbox contains helical gears, which mask the impulses generated by damaged gear teeth, and additionally the vibration signals are inherently impulsive. This impedes detecting damage in the gearbox.

The suitability of the SASE and the suitability of the proposed SMSE to detect localised damage are investigated in the next section.

### 4.2. Localised Gear Damage Investigation

**Figure 6:**
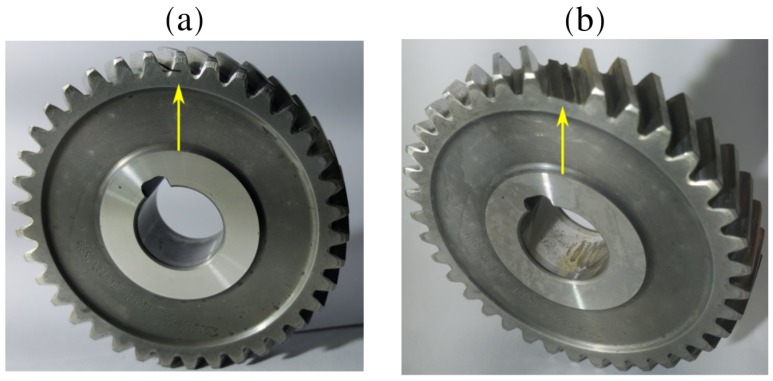
The gear with the seeded localised gear damage before the test is shown in (**a**) and the gear after the test was completed is shown in (**b**).

Localised gear damage was induced by seeding a slot in one of the teeth of the gear as shown in Figure 6a. This gearbox was operated under operating condition one in Figure 5, until the damaged gear tooth had failed. The gear after the completion of the test is shown in Figure 6b.

The raw vibration signal x(t) is order tracked by using the tacho signal generated with the zebra tape shaft encoder and the optical probe to obtain an angle-domain representation of the signal x(φ). Two-hundred measurements spaced over the life of the gear are investigated in this section.

**Figure 7:**
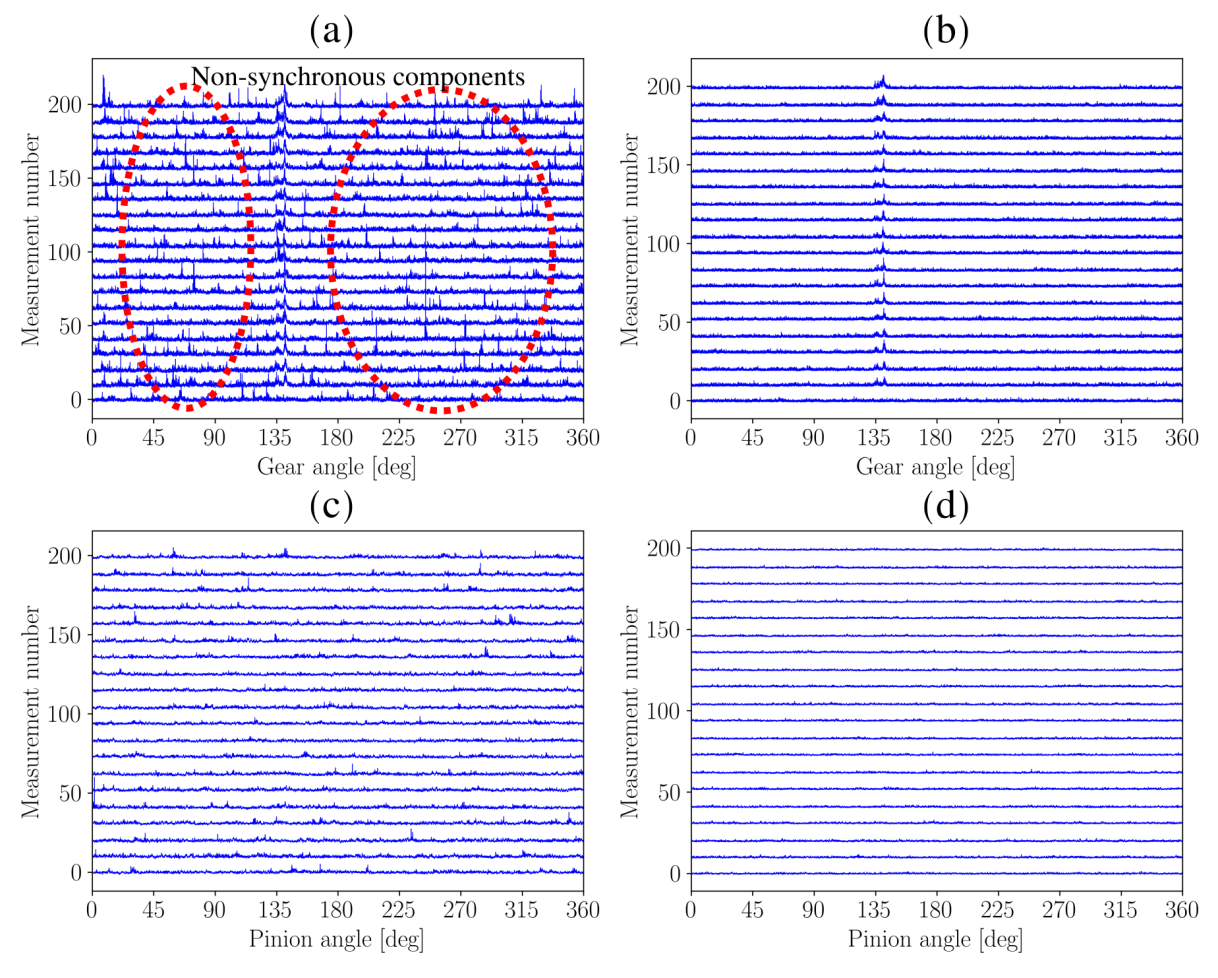
The Synchronous Average of the Squared Envelope (SASE) and the Synchronous Median of the Squared Envelope (SMSE) are shown for twenty of the two-hundred measurements for the gearbox that had a gear with localised damage and a healthy pinion. The red circles in (a) highlight the dominant non-synchronous components. (**a**) Damaged gear: SASE; (**b**) Damaged gear: SMSE; (**c**) Healthy pinion: SASE; (**d**) Healthy pinion: SMSE.

The SASE and the SMSE are calculated for the damaged gear, by using Equation (9) with $\Phi_{i}=1$ shaft rotation, and for the healthy pinion Equation (9) was used with $\Phi_{i}=0.54054$ shaft rotations, and presented in Figure 7. Even though two-hundred measurements are considered in this section, only twenty signals, evenly spaced over the life of the gear, are shown in Figure 7 to ensure that the results are easy to interpret.

The SASE for the gear in Figure 7a contains impulses scattered randomly over the rotation of the gear and gear damage that is located in the vicinity of 135 degrees. The aforementioned impulses are not related to the health of the gears and therefore impede the fault detection process. The only reason why it is possible to identify the gear damage at 135 degrees is because the measurements are aligned, which ensures that the position of the gear damage is the same between the different measurements. However, in applications where tacholess order tracking methods are used [37,38,39], it is not easy to align the different measurements and therefore the gear damage may be perceived as part of the random noise.

The SMSE is calculated with Equation (10) and presented in Figure 7b. The SMSE performs significantly better than the SASE, because the gear damage can clearly be seen at approximately 135 degrees, while the impeding impulses seen in the SASE are completely attenuated. The superiority of the SMSE over the SASE is further emphasised by the results of the pinion seen in Figure 7c,d; the impulses seen in SASE are not related to the condition of the pinion and make it difficult to determine its condition. In contrast, the SMSE of the pinion does not contain any evidence of damage and therefore provides the correct representation of the condition of both the gear and the pinion.

**Figure 8:**
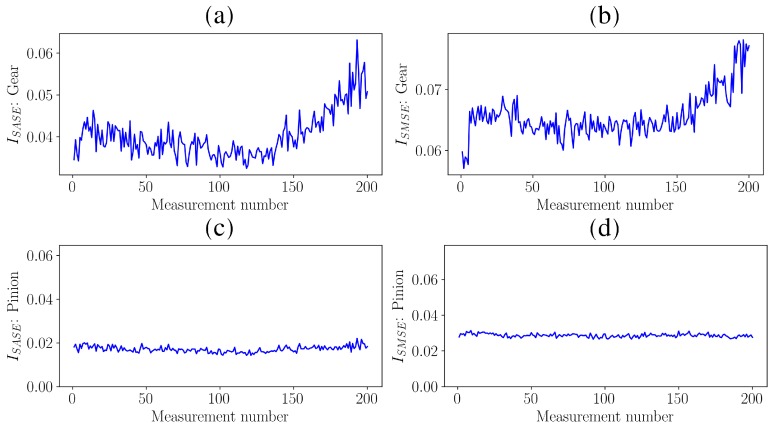
The metric calculated with Equation (11) with the SASE and the metric calculated with Equation (12) with the SMSE are calculated for two Φ cases. (**a**) SASE of the Gear (Φ=1.0 shaft rotation); (**b**) SMSE of the Gear (Φ=1.0 shaft rotation); (**c**) SASE of the Pinion (Φ=0.54054 shaft rotations); (**d**) SMSE of the Pinion (Φ=0.54054 shaft rotations).

The metrics using the SASE in Equation (11) and SMSE in Equation (12) are presented in Figure 8 for the gear and the pinion.

The metrics obtained with the SASE and SMSE of the gear shown in Figure 8a,b allow the degradation of the gear to be detected over time. The performance of the SASE indicator is attributed to the fact that the characteristics of the impulsive noise remained the same over measurement number, i.e., $\mu_{2}$ in Equation (5) was constant. However, it is conceivable that the characteristics of the impulsive noise could change over measurement number and would therefore lead to confusing metrics that make the fault diagnosis process more difficult. The pinion was relatively constant for both methods, which is correct. In the next investigation, it is shown that the condition indicator of the SASE could change as other mechanical components degrades as well.

### 4.3. Distributed Gear Damage Investigation

**Figure 9:**
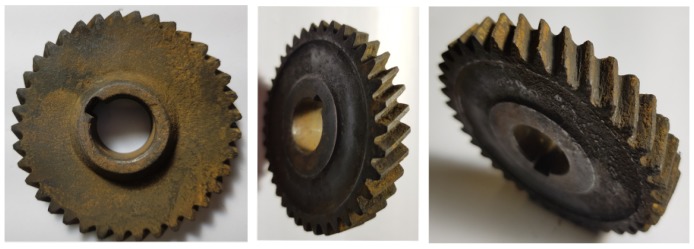
The gear with distributed damage before the test.

In the second investigation, distributed gear damage was induced on the gear by leaving the gear in a corrosive environment for approximately 1.5 years, with the result shown in Figure 9.

**Figure 10:**
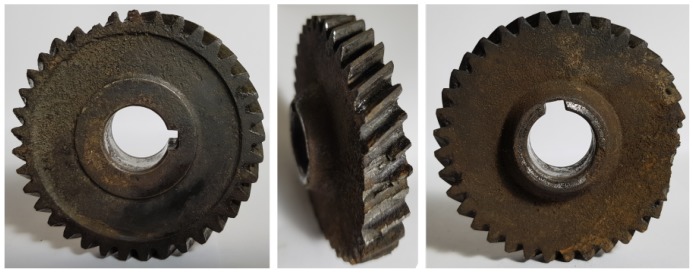
The gear with distributed damage after the test was completed.

This gear was operated for approximately 8 days with the operating conditions shown in Figure 5 whereafter the experiment was stopped due to excessive vibration. Three-hundred-and-twenty (320) measurements were acquired during the test, with the condition of the gear after the test shown in Figure 10.

The excessive vibration was caused by the failure of one gear tooth and the significant damage of two adjacent gear teeth. The pinion was again in a healthy condition for the duration of the test.

**Figure 11:**
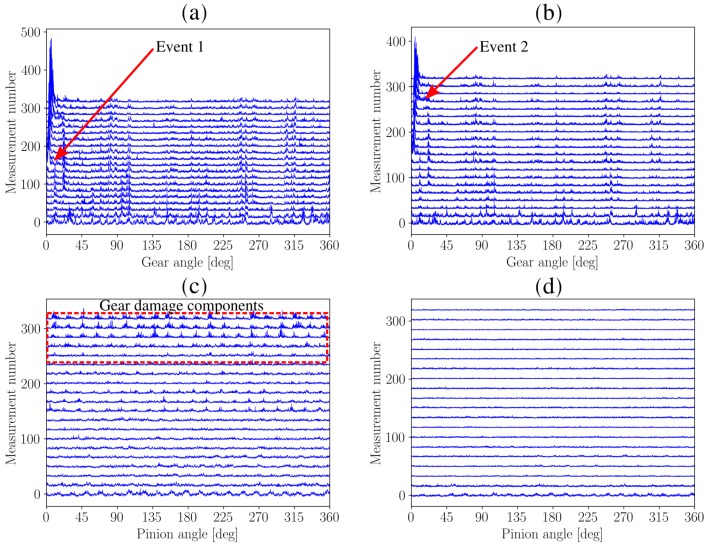
The Synchronous Average of the Squared Envelope (SASE) and the Synchronous Median of the Squared Average (SMSE) are presented for the gear and the pinion over twenty measurements of the distributed gear dataset. The twenty measurements were approximately equally spaced over the life of the gear. (**a**) Damaged gear: SASE; (**b**) Damaged gear: SMSE; (**c**) Healthy pinion: SASE; (**d**) Healthy pinion: SMSE.

The same procedure is followed as Section 4.2. Firstly, the vibration signals are order tracked, whereafter the SASE and SMSE are calculated for the gear and the pinion of the test gearbox. The SASE and SMSE of the gear and the pinion are presented in Figure 11. Only twenty of the 320 measurements are presented in the figure to ensure that it is easy to interpret.

The SASE and SMSE of the gear and the pinion perform very similarly as seen in Figure 11a,b. Much impulsive components can be seen over the rotation of the gear, e.g., between 45 and 90 degrees. This is attributed to the fact that distributed gear damage is present. At approximately the 150th measurement number, i.e., in the middle of the measurement number axis in Figure 11a,b, an event can be seen at approximately 0 degrees. A very large spike occurs which indicates that a gear tooth became severely damaged or has failed. This component also becomes more broad over measurement number which is indicative that adjacent teeth are potentially damaged as well.

The benefits of using the SMSE over the SASE is highlighted when investigating the results of the pinion in Figure 11c,d. The magnitude of the SASE of the pinion in Figure 11c contains very impulsive information which increases over measurement number. This is attributed to the SASEs sensitivity to the gear damage components, i.e., the SASE is not robust to non-synchronous impulsive components. The implication of this is that the condition of the pinion can be interpreted as becoming worse over measurement number. The SMSE in Figure 11b delivers completely different results; the first measurement contains some impulsive information which is attributed to wear in process of the gears, however, after this it can be seen that the SMSE of the pinion does not contain any impulsive information and is very uniform over measurement number. This means that the SMSE of the pinion is unaffected by the severely damaged gear and therefore provides a reliable representation of the condition of the pinion.

**Figure 12:**
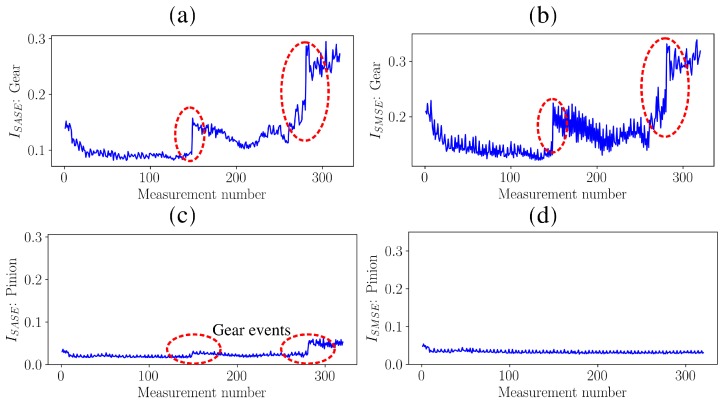
The metrics based on the Synchronous Average of the Squared Envelope (SASE) and the metrics based on the Synchronous Median of the Squared Envelope (SMSE) are presented for the damaged gear, which transitioned between the conditions shown in Figure 9 and Figure 10, and a healthy pinion. (**a**) Damaged gear: SASE; (**b**) Damaged gear: SMSE; (**c**) Healthy pinion: SASE; (**d**) Healthy pinion: SMSE.

These results are corroborated when investigating the metrics over measurement number in Figure 12.

The metrics of the gears, shown in Figure 12a,b for the SASE and the SMSE respectively, are able to detect the wear-in that occurred at the first few measurements due to the potential improvement of the corrosive surface of the gear. At measurement number 148, an event, indicated by Event 1 in Figure 12, occurred which resulted in a significant discontinuity in the metrics associated with the gear. When combining this information with the results in Figure 11a,b, this discontinuity is attributed to the sudden failure of a gear tooth. At measurement number 280, another event, indicated by Event 2 in Figure 12, occurred which is attributed to the failure of the adjacent teeth.

The SASE metric of the pinion in Figure 12c has a very similar behaviour of the gear; it is influenced by the condition of the gear and therefore is dependent on the measurement number. Hence, the SASE is unreliable for performing condition monitoring on gearboxes. The SMSE metric of the pinion in Figure 12d is unaffected by the changes in the condition of the gear and therefore remains relatively constant over measurement number. This correctly indicates that the pinion was healthy for the entire duration of the test and therefore the SMSE provides a more reliable estimate of the individual mechanical components under consideration. An example of the practical implication of diagnosing the condition of the gears incorrectly is that the maintenance department may order pinions and gears from the suppliers, while only gears are necessary. This can have significant financial implications when large gearboxes found in the power generation and mining industries are monitored.

**Figure 13:**
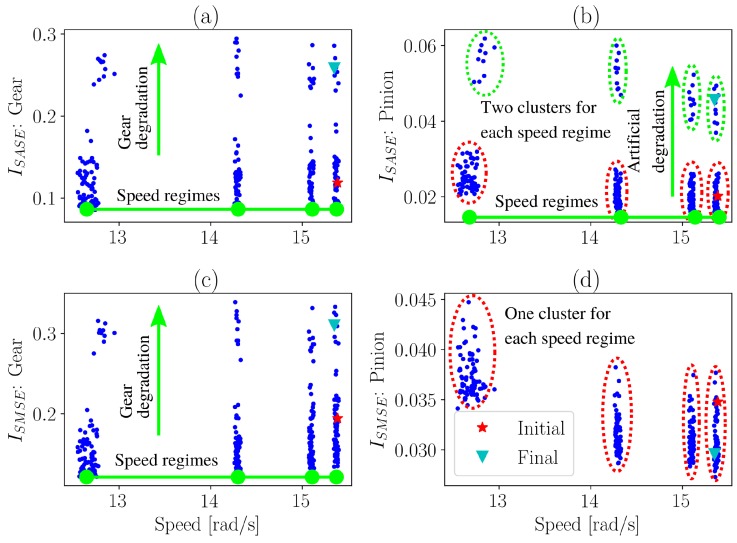
The condition indicator is presented over the average rotational speed for the different metrics and gears, i.e., it is the conditional condition indicator discussed in Section 2.4. One of the initial measurements as well as one of the final measurements of the test are highlighted to show how the data changes as the gear deteriorates. The clusters in (**b**,**d**) highlights that the SASE forms two clusters and the SMSE forms one cluster for a specific speed range. (**a**) Damaged gear: SASE; (**b**) Healthy pinion: SASE; (**c**) Damaged gear: SMSE; (**d**) Healthy pinion: SMSE.

The benefits of using the SMSE instead of the SASE are highlighted in Figure 13 where the condition indicators are presented over the rotational speed of the gearbox. The same presentation could not be performed for the localised gear damage experiment, since the gearbox was operating under the same time-varying operating conditions (i.e., OC: 1 in Figure 5) for each measurement.

This representation in Figure 13 allows the condition of machines to be inferred under time-varying operating conditions [4]. The four clusters on the rotational speed axis are attributed to the four operating conditions of the gearbox (i.e., see Figure 5).

Since the SASE does not provide a reliable estimation of the condition of the gearbox, two distinct clusters are formed when investigating the pinion for a specific operating condition state. This indicates that as the condition of the gear deteriorates, the condition indicator of the pinion erroneously indicates that the pinion is deteriorating as well. In contrast, the SMSE makes it possible to distinguish between the damaged gear and the healthy pinion. The condition indicator in Figure 13d is sensitive to operating conditions, however, when using the conditional representation in Figure 13, i.e., presenting the condition indicators against the operating conditions, the condition indicator becomes much more robust to changes in the operating conditions of the machine. Hence, the SMSE is a very simple method improvement to the SASE for performing gearbox fault diagnosis under time-varying and non-Gaussian noise conditions.

## 5. Conclusions

In this work, the synchronous median of the squared envelope is proposed and compared to the synchronous average of the squared envelope. It is emphasised throughout this paper that even though the change from the mean statistic to median statistic is subtle, it has significant benefits for condition monitoring. It is specifically shown that the synchronous median of the squared envelope can detect the presence of damage and is robust to impulsive noise and the impulses generated by other damaged components, which will be encountered in typical condition monitoring applications. As a result, the proposed synchronous median of the squared envelope can be used to reliably estimate the condition of the components-of-interest and can therefore assist with the maintenance decision making process. In future investigations, we will consider the suitability of the proposed method on other faults (e.g., bearing faults) under impulsive and time-varying operating conditions.

## Appendix A. The Multimodality of Synchronous Data

The potential multimodality of the synchronous data is illustrated in Figure A1. The data in Figure A1 can, for example, simulate the case where a component is damaged with a cyclic period of Φ=4 samples.

If the period of the synchronous data is Φ=3 or Φ=5, the larger amplitudes due to the damaged component are non-synchronous and result in the synchronous data to display multimodal characteristics. This phenomenon can be captured by a mixture model and therefore it is appropriate to use a mixture model to investigate the robustness of the metrics.
